# A Novel Signal-Amplified Immunoassay for the Detection of C-Reactive Protein Using HRP-Doped Magnetic Nanoparticles as Labels with the Electrochemical Quartz Crystal Microbalance as a Detector

**DOI:** 10.1155/2013/482316

**Published:** 2013-02-21

**Authors:** Ning Gan, Ping Xiong, Ji Wang, Tianhua Li, Futao Hu, Yuting Cao, Lei Zheng

**Affiliations:** ^1^The State Key Laboratory Base of Novel Functional Materials and Preparation Science, Faculty of Material Science and Chemical Engineering, Ningbo University, Ningbo 315211, China; ^2^Faculty of Mechanical Engineering and Mechanics, Ningbo University, Ningbo 315211, China; ^3^Department of Laboratory Medicine, Nanfang Hospital, Southern Medical University, Guangzhou, Guangdong 510515, China

## Abstract

A novel horseradish peroxidase- (HPR-) doped magnetic core-shell Fe_3_O_4_@SiO_2_@Au nanocomposites (Fe-Au MNPs) were employed on immunoassay for the determination of C-reactive protein (CRP) based on a electrochemical quartz crystal microbalance detector (EQCM). Firstly, the secondary CRP antibody and HRP were both immobilized on the Fe-Au MNPs (Fe-Au MNPs-anti-CRP2/HRP) as a signal tag. Secondly, the above tag and the primary antibody (anti-CRP1) in the bottom of 96-well microtiter plate were employed to conjugate with a serial of CRP concentrations to produce a sandwich immunocomplex. Thirdly, the immunocomplex solution was subsequently exposed to 3, 3′-diaminobenzidine (DAB) in the presence of H_2_O_2_, resulting in an insoluble product. When the precipitation solution was dripped on EQCM, it can achieve a decrease of frequency of crystal (Δ*f*). The amount of Δ*f* was proportional to (CRP) from 0.003 to 200 ng mL^−1^ with a low detection limit of 1 pg mL^−1^. Compared with the enzyme-linked immunosorbent assay (ELISA), the immunoassay shows greatly improved sensitivity due to the significant amount of HRP labeled on signal tag. It also has good specificity and low sample consumption, which is expected to be a benefit for the CRP screening in early diagnosis of cardiovascular disease.

## 1. Introduction

Cardiovascular disease (CVD) is a highly lethal disease. Thus, there must be excellent candidates for the rapid and inexpensive diagnosis of CVD [1]. C-reactive protein (CRP) is the most widely used as a CVD marker for the early diagnosis of the disease [2]. Recently, the usual way to predict CVD is detecting the concentration of CRP in serum. Ultrasensitive methodologies for detecting free CRP in human serums are required. The most common approaches in analyzing CRP are enzyme-linked immunosorbent assay (ELISA), immunofluorescence, and so forth [3]. However, most of usual methods are time consuming, labor intensive, and hazardous to health or require highly qualified personnel and sophisticated instrumentation [4–6].

Electrochemical detection is attracting more attention due to its low cost, wide dynamic concentration response range, versatility, simple instrumentation, stability, and high sensitivity [7]. Moreover, it is not affected by the sample components that might interfere with spectroscopic detection, such as particles, chromophores, and fluorophores [8]. Thus, the measurements can be made on blood samples without interference from red blood cells, hemoglobin, or other proteins. Among the electrochemical sensors, the electrochemical immunoassays that capitalize on the selectivity of antigen-antibody reactions have excellent detection limits. Quartz is one member of a family of crystals that experience the piezoelectric effect. The quartz crystal microbalance (QCM) can acquire an ultratrace mass per unit area by measuring the change in frequency of a quartz crystal resonator [9, 10]. The QCM typically consists of a thin disk of an AT-cut quartz crystal with circular metal electrodes on both surfaces. The QCM has been widely used to monitor the thickness change of film deposition according to the Sauerbrey equation [9] which relates the decrease in frequency Δ*f* of the QCM and the added mass Δ*m* of the thin film rigidly coupled to the crystal surface: $\Delta f=-(2f_{0}^{2}/A\sqrt{\rho_{q}\mu_{q}})\Delta m$, where *f*_0_ is the crystal fundamental frequency, *A* is the piezoelectric active area, and *ρ*_*q*_ and *μ*_*q*_ are the density (2650 kgm^−3^) and the shear modulus (2.94 × 10^10^ Nm^−2^) of the AT-cut quartz, respectively. QCM can also be used to investigate interactions between biomolecules, such as antigen and antibody. By measuring the change of frequency (Δ*f*) after the immunocomplex formed on the electrode, the signal can help to analyze the amount of antigen [11]. Furthermore, the QCM immunosensor is also a kind of label-free biosensor in immunoassays. Herein, the electrochemical quartz crystal microbalance (EQCM) was employed as the real-time transducer for detecting CRP in immunoassay.

Antibody immobilization is vital in successful development of an EQCM immunoassay, and the present immobilization methods, such as chemical modification, self-assembly, or physical absorption, are usually quite complex and liable to make the antibody deactivate in real application [12]. So if the antibody modification procedure can be excluded, there will be a good prospect for the method. Moreover, some of the enzyme-catalyzed products in sandwich ELISA were precipitated, so it can be directly measured on EQCM electrode. 3,3′-Diaminobenzidine (DAB) is usually employed as substrate in amplification of signal [13]. The HRP can biocatalyze DAB in the presence of H_2_O_2_, resulting in an insoluble product on the electrode surface, to achieve an obviously decreased frequency. The above detection principle has been widely used in the development of novel EQCM biosensor [14]. If elevating the amount of above insoluble catalyzed precipitates accumulated on EQCM transducers, the sensitivity of the immunoassay can also be greatly improved [15]. 

Signal amplification is the most important strategy that has been extensively used for the development of ultrasensitive immunoassay [16]. The use of nanomaterials as signal amplifiers is of particular interest in biosensor design, due to their outstanding optical, electronic, and biocompatible performance [17]. In the past decades, magnetic nanoparticles (MNPs) have attracted wide interests due to their unique features, such as good magnetic separation, thermal stabilities, and particle morphology [18]. Thus, MNPs take advantage over the convenient nanoparticles, and they are more suitable for usage in biology and medicine. Furthermore, superparamagnetic iron oxide nanoparticles, such as Fe_3_O_4_ nanoparticles (Fe_3_O_4_ NPs), have been functionalized with many different biological shells for interaction with biological molecules or cells so as to separate them by a magnetic field [19]. It is well known that nanogold particles (AuNPs) possess the property of high stability and the capacity to combine with amino or mercaptol group in biomolecules. Thus, combining Fe_3_O_4_ magnetic nanoparticles with silicon dioxide and AuNPs shell (Fe_3_O_4_@SiO_2_@Au) will have great potential application in biotechnology [20].

The most widely used synthesized method for Fe_3_O_4_@SiO_2_@Au core-shell magnetic nanoparticles was self-assembly. In this method, two kinds of nanoparticles were linked by coupling agent to form a strong chemical bond. The composites became more stable by employing this method because the magnetic particles were coated with a large amount of free amine group (–NH_2_) in the SiO_2_ shell with 3-aminopropyhriethoxysilane (APTS) which has been found to exhibit a strong binding force to colloid Au. In this study, Fe_3_O_4_ magnetic particles fictionalization of –NH_2_ was prepared firstly, then AuNPs magnetic composites particles were self-assembled on Fe_3_O_4_ NPs [21]. AuNPs have the capability to bind with the –SH in the biomolecules. The secondary antibody and HRP can both be immobilized on Fe_3_O_4_@SiO_2_@Au to synthesize the signal tag (Fe-Au MNPs-anti-CRP2/HRP). The antibody on the tag can capture CRP in solution, while HRP can catalyze DAB to amplify the change of frequency signal (Δ*f*). Thus, the immunoassay for CRP can be acquired.

Herein, CRP in serum is selectively captured by the primary antibody of CRP (anti-CRP1) deposited on the 96-well pore and then recognized by Fe-Au MNPs-anti-CRP2/HRP signal tag. The bound HRP will catalyze the H_2_O_2_-induced oxidation of DAB substrate to form an insoluble precipitation [22, 23] on the crystals, thus causing frequency changes (Δ*f*) which are proportional to the concentrations of targeting CRP. The main advantage of this enzyme-catalytic precipitation protocol lies in that the accumulation of an insoluble product on the crystals can lead to greatly increased mass changes as reflected by Δ*f*. It can achieve much higher detection sensitivity than some traditional ELISA methods [24] with mass changes directly originating from the large amount of HRP enzyme-labeled on the magnetic signal tag. In addition, this strategy combines the merits of electrochemical method and traditional ELISA method and smoothes away the cumbersome electrode modification process in electrochemical immunoassay.

## 2. Experimental Section

### 2.1. Chemicals and Materials

HRP-labeled monoclonal mouse anti-human CRP and CRP test kit were obtained from Sigma and Santa Cruz Co. Ltd. (USA). DAB and HRP (EC 1.11.1.7, RZ > 3.0, *A* > 250 U/mg) were purchased from Aldrich (USA). Tetraethylorthosilicate (TEOS), APTS, hydrogen tetrachloroaurate (III) tetrahydrate (HAuCl_4_·4H_2_O), H_2_O_2_, and bovine serum albumin (BSA) were obtained from Sinopharm Group Chem. Re. Co., Ltd. (Shanghai, China). All reagents used were of analytical-reagent grade, and all solutions were prepared with double-deionized water. Phosphate buffer saline (PBS, 0.1 M) of various pH was prepared by mixing the stock solutions of NaH_2_PO_4_ and Na_2_HPO_4_. The washing buffer was prepared with 0.02 mol/L PBS (pH 7.2) containing 0.5% BSA, 0.25% Tween-20, and 0.15 mol/L NaCl.

### 2.2. Apparatus

CHI 400 A electrochemical analyzer, crystals (9 MHz, gold electrodes), and QCM cell system were bought from CHI Instruments (Shanghai Chenhua Co., Ltd., China), in which the QCM cell consists of a detection cell and Ag/AgCl reference electrode and Pt wire counter electrode. The crystals were washed in an ultrasonic cleaner (B2000, Branson Co., Shanghai, China). The morphology of the nanoparticles was characterized by an H-7650 transmission electron microscope (TEM, Hitachi, Japan) and an S3400N scanning electron microscope (SEM, Hitachi, Japan). UV-Vis spectra were recorded with a TU-1901 spectrophotometer (Beijing Purkinje General Instrument Co., Ltd., China). 

### 2.3. Self-Assembly of Nano-Au on Fe_3_O_4_(Fe_3_O_4_@SiO_2_@Au)

The Fe_3_O_4_ NPs synthesized according to reference (0.3 g) [19] were dispersed in 20% ethanol (50 mL) by ultrasonic and APTS (0.4 mL) was added dropwise. The mixture was stirred at room temperature for 7 h. The desired aminated magnetic particles were afforded as light brown suspension. Uncoated Fe_3_O_4_ was deleted by means of washing the mixture with 0.1 mol/L HCl (six times, 4 h). Then the suspension was prepared to 1 g/L Fe_3_O_4_ with ethanol. The colloid Au (2.5 g/L, 10 mL) was added to the above prepared amination Fe_3_O_4_ NPs (25 mL) at room temperature with a stirring speed at 170 r/min. After stirring for 12 h, the excess Fe_3_O_4_ NPs were removed by addition of 0.1 mol/L HCl, and the precipitate was washed with distilled water until the eluted water became neutral (pH = 7.0). The ratio of Fe_3_O_4_@SiO_2_ and AuNPs (Fe_3_O_4_@SiO_2_/AuNPs) was 1 : 1.2. The concentration of Fe_3_O_4_@SiO_2_@Au was about 2 mg/mL.

### 2.4. Preparation of Fe-Au MNPs-Anti-CRP2/HRP Nanobioconjugation as Signal Tag

**Figure 1:**
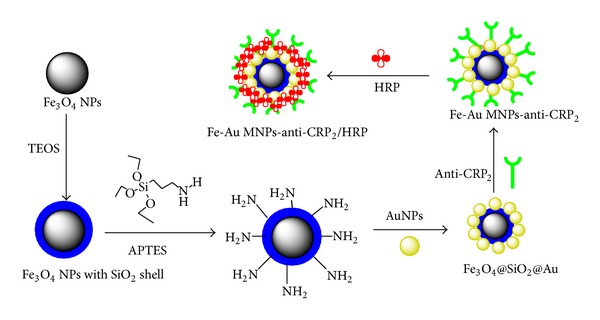
Schematic of the preparation of Fe-Au MNPs-anti-CRP2/HRP conjugation.

The procedure to prepare *Fe-Au MNPs-anti-CRP2/HRP* was shown in Figure 1. About 1.0 mL of Fe_3_O_4_@SiO_2_@Au nanoparticles suspension was initially adjusted to pH 8.2 using Na_2_CO_3_, and then 10 *μ*L of the original anti-CRP2 (10 *μ*g/mL) was added into the mixture and incubated for 12 h at 4°C with slightly stirring. After magnetic separation, the obtained Fe-Au MNPs-anti-CRP2 conjugates can be easily acquired and then were incubated with 1.5 mL of 1 mg/mL HRP for 1 h to block the nonspecific sites on the uncovered surface of Fe_3_O_4_@SiO_2_@Au nanoparticles. Then also after magnetic separation, the synthesized Fe-Au MNPs-anti-CRP2/HRP bioconjugations were present in the precipitation, which can be stored in 2 mL of pH 7.4 PBS at 4°C when not in use. 

### 2.5. Immunoassay Procedures

**Figure 2:**
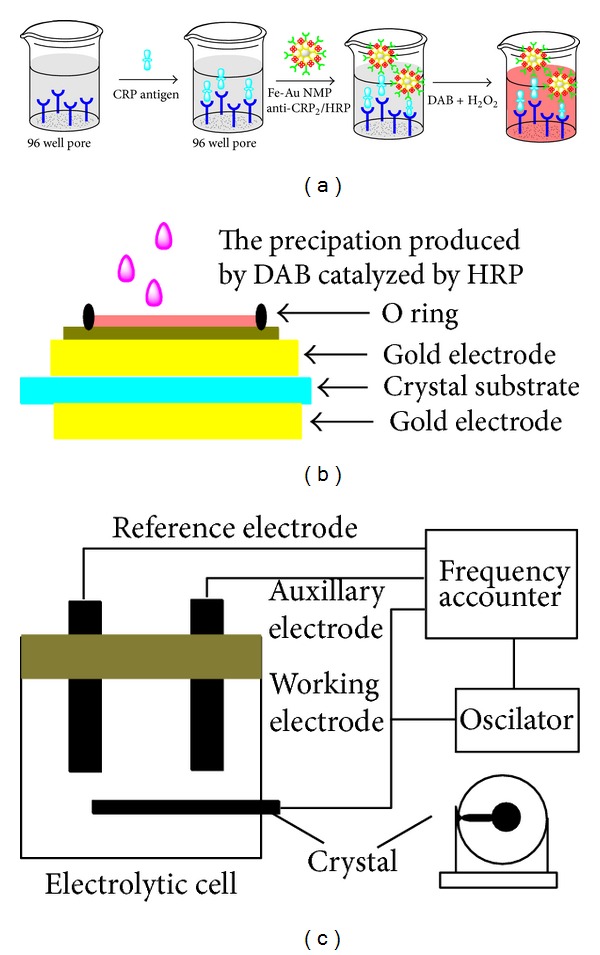
Schematic of the preparation of Fe-Au MNPs-anti-CRP2/HRP conjugation and the immunoassay procedure with the application of Fe-Au MNPs-anti-CRP2/HRP as the signal tag (a) and the EQCM's measurement steps (b) and detection system (c).

A schematic representation of the steps used to perform the electrochemical ELISA was shown in Figure 2(a). An anti-CRP precoated polystyrene 96-well microtiter plate was incubated with 100 *μ*L of different concentrations CRP for 30 min at 37°C. After incubation, the wells were washed six times with pH 7.4 PBS containing 0.05% (W/V) Tween-20. Then 100 *μ*L of Fe-Au MNPs-anti-CRP2/HRP conjugations (1 : 10 dilution with PBS containing 1 wt.% BSA) was pipetted into each well and incubated at 37°C for 30 min. After the wells were rinsed, 100 *μ*L of 10 mmol/L DAB and 3 mmol/L H_2_O_2_ mixture solution were added to each well, and the enzymatic reaction was allowed to proceed for 10 min at 37°C. The resulting reaction solution (20 *μ*L) was dropped onto EQCM electrode with 5 *μ*L PBS (pH 7.0, Figures 2(b) and 2(c)). After each detection, the electrode was instantaneously washed with PBS solution for 5 times to remove the precipitation solution. Because the precipitation by DAB was dropped on the electrode, it can be easily washed away; thus, the EQCM detector can be renewed.

## 3. Results and Discussion

### 3.1. Characterization of Fe-Au MNPs-Anti-CRP2/HRP Nanobioconjugation

**Figure 3:**
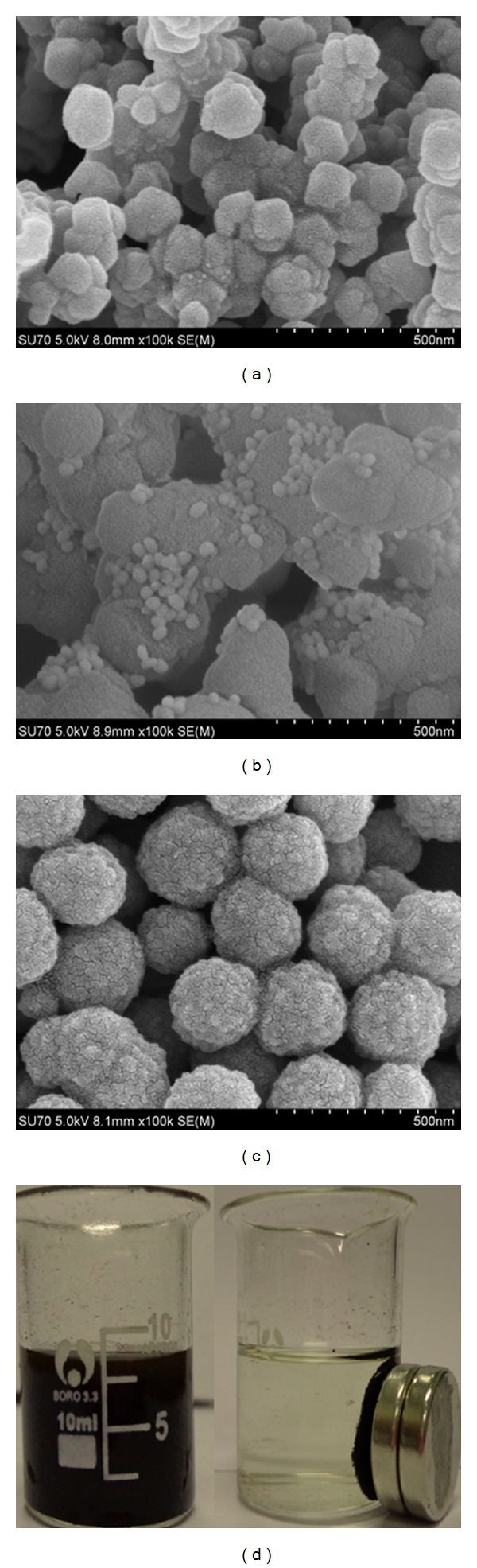
SEM images of (a) Fe_3_O_4_@SiO_2_, (b) Fe_3_O_4_@SiO_2_@Au, (c) Fe-Au MNPs-anti-CRP2/HRP signal tag, (d) c in absence (right) and presence (left) of magnetic field.

The core-shell Fe_3_O_4_@SiO_2_@Au nanoparticle was used to label anti-CRP2 and HRP. The SEM images showed that both amination Fe_3_O_4_-NH_2_ and Fe_3_O_4_@SiO_2_@Au nanoparticles were of well spherical structure and preferable monodispersity in size. The average diameter of Fe_3_O_4_-NH_2_ nanoparticles and core-shell Fe_3_O_4_@SiO_2_@Au nanoparticles were about 100 nm (Figure 3(a)) and 120 nm (Figure 3(b)), respectively, demonstrating that the Au shell was about 10 nm thick. As shown in Figure 3(c), the SEM spectrum of Fe-Au MNPs-anti-CRP2/HRP showed the particle was homogeneous and its diameter was enhanced to 250 nm. These proved that the large biomolecule of HRP and anti-CRP2 can be immobilized on Fe_3_O_4_@SiO_2_@Au nanoparticles. Once an external magnetic field was applied, the complex was attracted quickly toward the magnet and redispersed into water while removing the magnet, indicating Fe-Au MNPs-anti-CRP2/HRP signal tag has good paramagnetism (Figure 3(d)).

**Figure 4:**
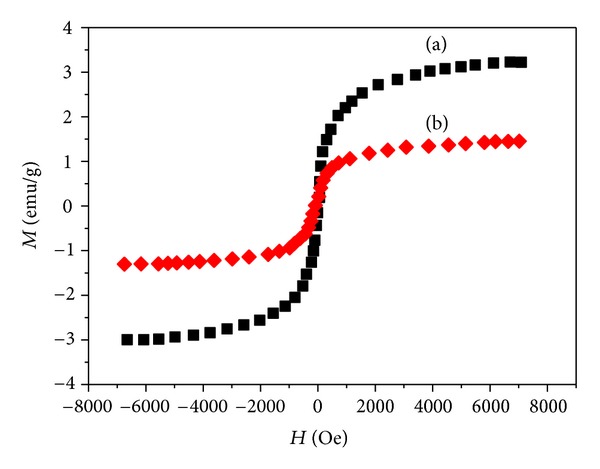
Hysteresis loops of magnetic silica nanospheres with (a) Fe_3_O_4_-NH_2_ and (b) Fe_3_O_4_@SiO_2_@Au at 300 K, respectively.

The magnetic properties of the nanoparticles are illustrated in Figure 4. It can be seen that a small coercivity or remanence existed around room temperature, indicating that the Fe_3_O_4_@SiO_2_ and Fe_3_O_4_@SiO_2_@Au have superparamagnetic properties, which explained the Fe_3_O_4_@SiO_2_@Au could be employed for magnetic separation. The saturation magnetization values for Fe_3_O_4_@SiO_2_ and Fe_3_O_4_@SiO_2_@Au were 3.34 emu/g and 1.29 emu/g, respectively. The reason for the saturation magnetization value of Fe_3_O_4_@Au being smaller than Fe_3_O_4_-NH_2_ is that the former has a larger shell of Au without magnetic features.

**Figure 5:**
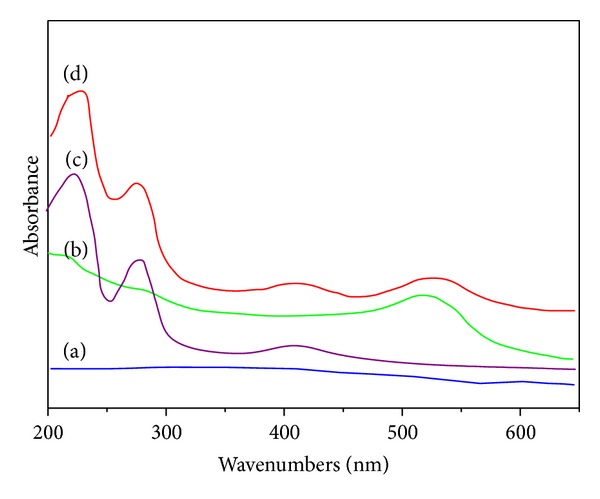
UV-Vis absorption spectrum of (a) Fe_3_O_4_@SiO_2_, (b) Fe_3_O_4_@SiO_2_@Au, (c) anti-CRP2/HRP, (d) Fe-Au MNPs-anti-CRP2/HRP conjugation.

UV-Vis absorption spectrometry was also employed to characterize the nanobioconjugation of Fe-Au MNPs-anti-CRP2/HRP. The Fe_3_O_4_ nanoparticles showed no characteristic absorption peak in the examined range from 200 to 650 nm (Figure 5(a)). When gold nanoparticles were deposited on the surface of Fe_3_O_4_ NPs, a new absorption band centered at 520 nm resulted from gold nanoparticles was observed (Figure 5(b)), indicating the Fe_3_O_4_@SiO_2_@Au nanoparticles were successfully prepared, which was in good agreement with the results of Lai et al. [20]. After the HRP and anti-CRP molecules being labeled onto the surface of the Fe_3_O_4_@SiO_2_@Au nanoparticles, the obtained Fe-Au MNPs-anti-CRP2/HRP nanobioconjugate exhibited two absorption peaks at 280 and 410 nm [21] (Figure 5(d)). The peak at 520 nm originated in Au peaks on the Fe_3_O_4_@SiO_2_@Au nanoparticles. The peak at 280 nm and 410 nm was respectively attributed to the absorption of anti-CRP2 and HPR as judged from Figure 5(c). On the basis of the above results, it can be concluded that Fe-Au MNPs-anti-CRP2/HRP nanobioconjugation was successfully prepared.

### 3.2. The Characterization of Signal Amplification by Fe-Au MNPs-Anti-CRP2/HRP_2_ by Different Labels

**Figure 6:**
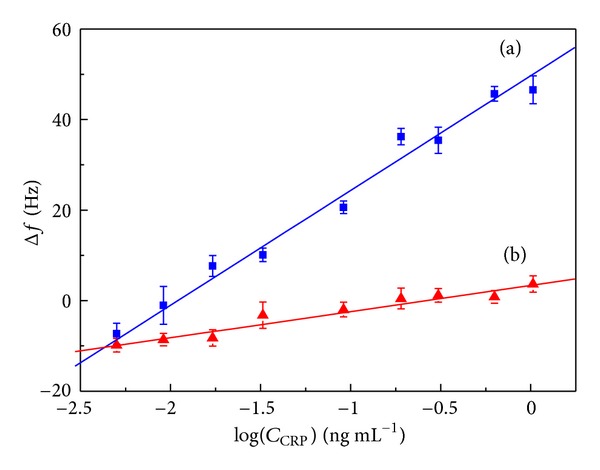
The calibration curve of the immunosensor with (a) Fe-Au MNPs-anti-CRP2/HRP and (b) HRP-anti-CRP2 signal tag.

To clarify the amplification effect of Fe-Au MNPs-anti-CRP2/HRP_2_ bioconjugation as the signal tag, we constructed two types of tags, including HRP-anti-CRP2 and Fe-Au MNPs-anti-CRP2/HRP_2_ for comparison. Different concentrations of CRP were used as an example for the evaluation of the signal response. The judgment was based on the slope of frequency change (Δ*f*) with the concentration. As shown in Figure 5, the use of Fe-Au MNPs-anti-CRP2/HRP_2_ as the detection antibody (Δ*f* = 24.1 Hz/log⁡⁡*C* A,Figure 6(a)) exhibited much higher signal response than that of applying HRP-AFP Ab2 (Δ*f* = 4.8 Hz/log⁡⁡*C*, Figure 6(b)). The sensitivity of the former is about 5-fold of the latter which meant that the immunosensor by Fe-Au MNPs-anti-CRP2/HRP_2_ can generate significant signal amplification than HRP-anti-CRP2. The reason might be that the amount of labeled HRP is much higher in Fe-Au MNPs-anti-CRP2/HRP_2_ detection antibody than in HRP-anti-CRP2. When one Fe-Au MNPs-anti-CRP2/HRP_2_ detection antibody reacted with the antigen, dozens of HRP can catalyze DAB to produce a large amount of precipitate in the present of H_2_O_2_.

### 3.3. Optimization of Experimental Conditions

The immunoassay conditions such as the amount of the Fe-Au MNPs-anti-CRP2/HRP-HRP and DAB on the electrode, pH of the supporting electrolyte, incubation temperature, and the incubation time can affect the ECL response of the immunosensor.

**Figure 7:**
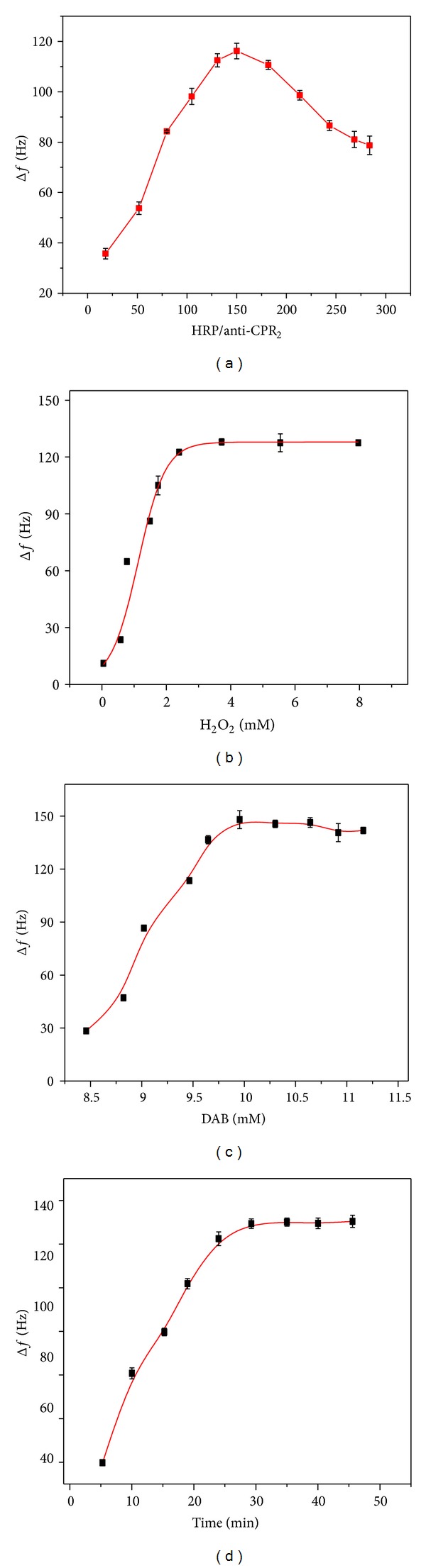
(a) Optimization of volume ratio of HRP to anti-CRP2 for preparation of Fe-Au MNPs-anti-CRP2/HRP. Effects of concentration of (b) H_2_O_2_, (c) DAB, and (d) incubation time on the signal response at the immunosensor.

The concentration of HRP and anti-CPR2 labeled on Fe_3_O_4_@SiO_2_@Au highly influences the performance of Fe-Au MNPs-anti-CRP2/HRP-HRP signal tag. To obtain a stable and sensitive immunoassay and maintain its precipitation forming ability, the concentration of HPR and anti-CPR2 was, respectively, set as 1 *μ*g/mL and 1 mg/mL. Then mix solution with different volume ratios of HRP and anti-CPR2 was labeled on Fe_3_O_4_@SiO_2_@Au to construct the signal tag for measurement. The results (Figure 7(a)) revealed that HRP and anti-CRP2 solution in the volume ratio of 150 : 1 was optimal. Further, based on the experimental results, 10 *μ*L of Fe-Au MNPs-anti-CRP2/HRP-HRP signal tag composite solution was selected as the optimal amount dropped on the electrode surface.

The concentrations of H_2_O_2_ and DAB are important parameters that influence the activity of HRP. Using 10.0 ng·mL^−1^ CRP as a model, we monitored the effect of concentrations of H_2_O_2_ and DAB on the current response of the electrochemical ELISA. The reduction peak current of the enzymatic oxidation product increased with the increasing concentrations of H_2_O_2_ (Figure 7(b)) and DAB (Figure 7(c)) maintained the maximum value at higher concentrations. Afterward, the enzymatic reaction rate depended on the amount of the labeled HRP. Therefore, the optimal concentrations of 10 mmol/L for DAB and 3 mmol/L for H_2_O_2_ were used for the immunosensor. 

The effects of incubation temperature and incubation time on the EQCM response of the immunosensor were also investigated. The results revealed that Δ*f* increased with the increase of incubation temperature from 20 to 45°C and a maximum Δ*f* was obtained at 37°C.  Figure 7(d) shows that Δ*f* increased with the increase of incubation time and reached a plateau at 30 min. Therefore, 37°C and 30 min were selected as the optimum incubation temperature and time in this study.

### 3.4. Analytical Performance

Under the optimal conditions, the electrochemical ELISA was carried out to analyze various concentrations of CRP solution and the EQCM response was recorded. The Δ*f* of EQCM response increased with the increasing CRP concentration. The linear range was 0.003~200 ng/mL with a limit of detection (LOD) of 1 pg/mL.

### 3.5. Specificity, Reproducibility, and Stability

To evaluate the specificity of the proposed electrochemical ELISA for CRP detection, various biomarkers including carcinoma antigen 125 (CA 125), carcinoembryonic antigen (CEA), human IgG (HIgG), and prostate-specific antigen (PSA) were tested. The electrochemical signals were recorded in 10 ng/mL CRP with and without the interfering agents. No significant difference of currents was observed in comparison with the result obtained in the presence of only CRP, indicating that the specificity of the proposed electrochemical ELISA was acceptable. The reproducibility of the immunoassay was evaluated by using the coefficients of variation of intra-assays and interassays. Taking 10 ng·mL^−1^ CRP for example, the intra-assays and interassays of the electrochemical were evaluated using one electrochemical ELISA for five repeat assays and five copies of electrochemical ELISA for one time assay. The coefficients of variation of the intra-assay and interassay were 3.4% and 2.4%, respectively. Thus, the reproducibility of the electrochemical ELISA is satisfactory. The stability of the synthesized nanobioconjugate of Fe-Au MNPs-anti-CPR2/HRP was also examined. When not in use, it could be stored in pH 7.0 PBS for at least 2 weeks without obvious signal change. Moreover, it retained 94.2% of its initial response after a storage period of 3 weeks. The slow decrease in the current response may be attributed to the gradual deactivation of the immobilized biomolecules on the surface of the nanoparticles. 

### 3.6. Determination of CRP in Human Serum Samples

In order to investigate the possibility of the developed method for clinical analysis, some of patient human serum samples were examined by the developed method and the results were compared with the classical ELISA method. The concentration of CRP in three samples was determined to be consistent with the results obtained by the classical ELISA method (Table 1), indicating that the proposed immunoassay could be satisfactorily applied to the clinical determination of CRP. In Table 1, comparison of CRP levels in patient human serum samples (*n* = 3) was determined using two methods.

## 4. Conclusions

In this study, an ultrasensitive electrochemical immunoassay was developed for the detection of CRP in serum by using a newly designed magnetic HRP-labeled signal tag. The synergistic applications of Fe_3_O_4_@SiO_2_@Au nanoparticles and HRP labeled anti-CRP antibodies have been verified to well circumvent the specific recognition of targeting CRP. H_2_O_2_ oxidization of the DAB substrate by HRP catalysis could produce an insoluble precipitation on the crystal resulting in greatly amplified QCM responses. The Fe_3_O_4_@SiO_2_@Au nanoparticle can provide a larger surface area for loading the enzyme-catalytic product in addition to serving as the selective sorbents. Moreover, DAB was verified as the ideal chromogenic substrate suitable for enzyme-catalytic precipitation. Ultrasensitive quantitative and rapid qualitative QCM analysis of the targets was achieved. This proposed QCM immunoassay is simple, of real-time, sensitive, and field applicable, holding great promise of being applied as an alternative tool for screening the concentration of CRP in CAD.

## Figures and Tables

**Table 1:** Comparison of CRP levels in patient human serum samples (*n* = 3) determined using two methods.

Serum samples	The developed method	The classical ELIAS
	concentration (ng·mL^−1^)	concentration (ng·mL^−1^)
Sample 1	8.12 ± 0.21	8.5
Sample 2	5.31 ± 0.41	5.5
Sample 3	21.1 ± 0.22	22
Sample 4	1.1 ± 0.03	ND

*ND: not detected.

## References

[1] McGill HC, McMahan CA, Zieske AW (2000). Associations of coronary heart disease risk factors with the intermediate lesion of atherosclerosis in youth. *Arteriosclerosis, Thrombosis, and Vascular Biology*.

[2] Kaptoge S, Di Angelantonio E, Lowe G (2010). C-reactive protein concentration and risk of coronary heart disease, stroke, and mortality: an individual participant meta-analysis. *The Lancet*.

[3] Swiatkiewicz I, Kozinski M, Magielski P (2012). Usefulness of C-reactive protein as a marker of early post-infarct left ventricular systolic dysfunction. *Inflammation Research*.

[4] Bisoendial RJ, Boekholdt SM, Vergeer M, Stroes ESG, Kastelein JJP (2010). C-reactive protein is a mediator of cardiovascular disease. *European Heart Journal*.

[5] Danesh J, Pepys MB (2009). Editorial: C-reactive protein and coronary disease: is there a causal link?. *Circulation*.

[6] Johnson A, Song QF, Ferrigno PK, Bueno PR, Davis JJ (2012). Davis Sensitive affimer and antibody based impedimetric label-free assays for C-reactive protein. *Analytical Chemistry*.

[7] Kumar D, Prasad BB (2012). Multiwalled carbon nanotubes embedded molecularly imprinted polymer-modified screen printed carbon electrode for the quantitative analysis of C-reactive protein. *Sensors and Actuators B: Chemical*.

[8] Male KB, Hrapovic S, Luong JHT (2007). Electrochemically-assisted deposition of oxidases on platinum nanoparticle/multi-walled carbon nanotube-modified electrodes. *Analyst*.

[9] Sauerbrey GZ (1959). Use of quartz vibration for weighing thin films on a microbalance. *Physik Journal*.

[10] Chen XJ, Wang YY, Zhou JJ, Yan W, Li XH, Zhu JJ (2008). Electrochemical impedance immunosensor based on three-dimensionally ordered macroporous gold film. *Analytical Chemistry*.

[11] Park JW, Kurosawa S, Aizawa H, Goda Y, Takai M, Ishihara K (2006). Piezoelectric immunosensor for bisphenol A based on signal enhancing step with 2-methacrolyloxyethyl phosphorylcholine polymeric nanoparticle. *Analyst*.

[12] Uludağ Y, Tothill IE (2010). Development of a sensitive detection method of cancer biomarkers in human serum (75%) using a quartz crystal microbalance sensor and nanoparticles amplification system. *Talanta*.

[13] Fu T, Wang H, Shen GL, Yu RQ (2006). An amplified piezoelectric immunosensor based on amplification of enzyme-catalyzed precipitation mass. *Chemical Journal of Chinese Universities*.

[14] Wang H, Wang J, Choi D, Tang Z, Wu H, Lin Y (2009). EQCM immunoassay for phosphorylated acetylcholinesterase as a biomarker for organophosphate exposures based on selective zirconia adsorption and enzyme-catalytic precipitation. *Biosensors and Bioelectronics*.

[15] Tang D, Li Q, Tang J, Su B, Chen G (2011). An enzyme-free quartz crystal microbalance biosensor for sensitive glucose detection in biological fluids based on glucose/dextran displacement approach. *Analytica Chimica Acta*.

[16] Magni F, van der Burgt YE, Chinello C (2010). Biomarkers discovery by peptide and protein profiling in biological fluids based on functionalized magnetic beads purification and mass spectrometry. *Blood Transfusion*.

[17] Wang J, Han HY, Jiang XC, Huang L, Chen L, Li N (2012). Quantum dot-based near-infrared electrochemiluminescent immunosensor with gold nanoparticle-graphene nanosheet hybrids and silica nanospheres double-assisted signal amplification. *Analytical Chemistry*.

[18] Hnaiein M, Hassen WM, Abdelghani A (2008). A conductometric immunosensor based on functionalized magnetite nanoparticles for E. coli detection. *Electrochemistry Communications*.

[19] Wang X, Huang S, Shan Z, Yang W (2009). Preparation of Fe_3_O_4_@Au nano-composites by self-assembly technique for immobilization of glucose oxidase. *Chinese Science Bulletin*.

[20] Lai CW, Wang YH, Lai CH (2008). Iridium-complex-functionalized Fe_3_O_4_/SiO_2_ core/shell nanoparticles: a facile three-in-one system in magnetic resonance imaging, luminescence imaging, and photodynamic therapy. *Small*.

[21] Gan N, Jin HJ, Li TH, Zheng L (2011). Fe_3_O_4_/Au magnetic nanoparticle amplifcation strategies for ultrasensitive electrochemical immunoassay of alfa-fetoprotein. *International Journal of Nanomedicine*.

[22] Inger V-L, Willem MA (2006). Site-directed immobilisation of antibody fragments for detection of C-reactiveprotein. *Biosensors and Bioelectronics*.

[23] Wolbink GJ, Brouwer MC, Buysmann S, Ten Berge IJM, Hack CE (1996). CRP-mediated activation of complement in vivo: assessment by measuring circulating complement-C-reactive protein complexes. *Journal of Immunology*.

[24] Levan-Petit I, Cardonna J, Garcia M (2000). Sensitive ELISA for human immunoglobulin D measurement in neonate, infant, and adult sera. *Clinical Chemistry*.

